# The Effects of COVID-19-Related Restrictions on Parkinson’s Disease Patients in Italy: Results of a Structured Survey

**DOI:** 10.3390/jcm11113007

**Published:** 2022-05-26

**Authors:** Stefano Martini, Luca Magistrelli, Francesca Vignaroli, Federico Colombatto, Cristoforo Comi, Marco Cosentino

**Affiliations:** 1 Center for Research in Medical Pharmacology, University of Insubria, 21100 Varese, Italy; stefano.martini@uninsubria.it (S.M.); marco.cosentino@uninsubria.it (M.C.); 2 Movement Disorders Centre, Neurology Unit, Department of Translational Medicine, University of Piemonte Orientale, 28100 Novara, Italy; magis.luca@gmail.com (L.M.); francescavignaroli@outlook.it (F.V.); federico.colombatto@hotmail.it (F.C.); 3 Neurology Unit, S. Andrea Hospital, Department of Translational Medicine, University of Piemonte Orientale, 13100 Vercelli, Italy; 4 Center for Research in Neuroscience, University of Insubria, 21100 Varese, Italy

**Keywords:** Parkinson’s disease, COVID-19, lockdown, quarantine

## Abstract

COVID-19 was first identified in China in late 2019 and spread globally, originating a pandemic. To limit the spreading of the virus, many countries, including Italy, introduced social distancing measures and limited human movement. The Italian government declared a lockdown of the whole country lasting about two months, and the introduced restrictive rules heavily impacted patients with chronic neurological diseases because of the reduced access to healthcare and community support services. In Parkinson’s disease, studies confirmed lockdown restrictions increase levels of psychological distress, impose limitations on physical activities, and cause a lack of clinical assistance. This study aims at investigating the impact of the pandemic during and beyond the lockdown period in such patients using an online survey. A total of 387 total patients accessed the survey and were asked about their personal experiences during and after lockdown. The results show a significant impact on people’s lives even months after lockdown restrictions were lifted, with a substantial and durable worsening in different aspects of daily life, heavily influenced by impaired access to health services—particularly physical therapies, including personal physical activity—and readily available clinical counselling, with an overall observation of worsening symptoms control. These aspects should be carefully considered in the assessment of global health care strategies to overcome the current pandemic and its broader effects.

## 1. Introduction

The coronavirus disease 2019 (COVID-19) is a respiratory illness caused by the SARS-CoV-2 virus, first identified in Wuhan, China, in late 2019 [1,2]. During the following months, SARS-CoV-2 spread globally, and on 11 March 2020, the World Health Organization declared the SARS-CoV-2 outbreak a pandemic [2,3]. To limit the spreading of the virus, many countries introduced a series of measures promoting social distancing and limiting human movement, both within and between different countries. Social distancing rules included working from home, closing restaurants and schools, and the interruption of non-essential activities. [2,3,4,5]. At the end of February, Italy was strongly hit by the SARS-CoV-2 outbreak, and on 8 March 2020, the Italian government declared a lockdown of the whole country until 4 May 2020 [2,6]. The COVID-19 pandemic and the related restrictive rules totally upset the lives of people. In light of this, patients with chronic neurological diseases suffered particularly because of reduced access to healthcare and community support services, especially because they need regular healthcare appointments and close contact with their caregivers [5,7,8]. For example, people affected by multiple sclerosis (MS) reported increased difficulties in daily life activities [4], and some MS patients self-discontinued their disease-modifying therapy (DMTs) due to fear of being infected by SARS-CoV-2 [9]. Amyotrophic lateral sclerosis (ALS) patients, who are in need of multidisciplinary care, also suffered greatly because of limited access to hospitals during lockdown [10]. For people with dementia, understanding the reasons behind the need for self-isolation might have been difficult, and a significant worsening of cognitive decline in these patients has been reported [11]. Moreover, for patients with dementia, a sudden change in daily activity can trigger agitation and aggressive reactions [12]. In regard to Parkinson’s Disease (PD), studies confirmed that lockdown restrictions severely affected PD patients. For example, the pandemic increased levels of psychological distress and imposed limitations on physical activities, which probably indirectly caused worsened motor symptoms (e.g., tremor, rigidity) [2,5,13]. Moreover, PD patients reported a lack of clinical assistance [2] and difficulties in obtaining PD medications [1,3,14]. Interestingly, as a consequence of social restrictions, these patients experienced a greater subjective worsening of their condition than patients affected by different chronic neurological diseases [15]. This different burden may be related to PD patients’ need for supportive therapies (such as neurorehabilitation, speech, and occupational therapies) in addition to pharmacotherapy, which were significantly reduced during the pandemic due to social restrictions. Indeed, other chronic neurological conditions that benefit from multi-disciplinary approaches showed a similar excess in unmet needs for urgent neurological care [15].

With this background, this study aimed to investigate the impact of COVID-19 on daily activities and the management of disease in PD patients during and beyond the lockdown period. While other studies evaluated the situation of PD patients during the lockdown, no information is currently available on long-lasting effects after the lifting of stricter restrictions. Such information could provide a useful insight into possibly overlooked and unmet needs in the PD population, allowing not only a more efficient and effective recovery but also better management of patients’ needs.

## 2. Materials and Methods

PD patients were invited to participate in an online survey, shared via social media, through patients’ associations, or by clinical neurologists during routine examinations in three different clinical settings. In particular, the survey’s web link was shared by five different groups among the “Insubria Parkinson Association” (Varese, Cassano Magnago, Groane, Legnano, and Novara), which includes patients living in a region in north-western Italy, across the Italian provinces of Varese, Como, Verbano-Cusio-Ossola, Novara, and Lecco; clinical neurologists working in Novara, Varese, and Tradate shared the web link with their patients, hailing from a similar geographical basin; social media posts were open to all.

The questionnaire was made available to participants after the lockdown period, between October 2020 (when the first one was collected) and April 2021. Participants were asked to fill in the questionnaire on their own, eventually relying on practical help from their caregivers but reporting their own personal experiences. A total of 387 patients accessed the survey, and 339 completed section A of the questionnaire, 276 completed section B, 260 completed section C, and 246 completed the whole questionnaire. The survey was conducted according to the Italian Code regarding the protection of personal data (http://garanteprivacy.it, accessed on 1 October 2020), and all the participants consented to the use of their information, provided in anonymous form, for the purposes of the present study. The web-based survey was available through the Survey Monkey platform (www.surveymonkey.com, accessed on 1 October 2020) and consisted of 27 questions divided into 4 sections:

- Opinion and perception of the epidemic (8 questions, 8 items).
- Personal experience and involvement in the epidemic (7 questions, 20 items).
- Current situation (3 questions, 11 items).
- Personal data (9 questions, 11 items).

Survey items related to disability and personal data were intentionally positioned at the end of the survey to maximize participants’ compliance. The whole survey was presented in Italian. See Supplementary Material 1 for collected data. The English translation of questionnaire items is reported in Table 1.

Most items are based on a 5-point Likert scale, while some of the questions required a yes/no answer, and a few items allowed participants to enter free text. Such text was collected and used to better understand participants’ opinions. No results relied only on free-text items due to excessive variability and the possibility of a misinterpretation of participants’ answers. It is worth noting that the questionnaire was developed in collaboration with the patients’ associations, which also contributed to the dissemination of the survey. Thus, some of the questionnaire items are only marginally related to the specific aim of this research and were included to obtain a better insight into general data about the participants’ situation and characteristics.

## 3. Results

A total of 387 PD patients were invited to answer our web-based questionnaire. Section A was completed by 339 patients, section B by 276, section C by 260, and section D by 246 (63.6%). For each of the four parts, we considered only the individuals who entirely completed the section.

### 3.1. Section A—Opinion and Perception of the Epidemic

This first section was completed by 339 individuals. In this section of our survey, we evaluated PD patients’ perception of the COVID-19 epidemic.

Results from this section are schematically presented in Table 2.

Most of the participants felt quite worried about the COVID-19 outbreak (question A1), social distancing, and crowded places (question A3), in particular hospitals and ambulatories (question A6). Moreover, most of our patients considered their risk of contracting SARS-CoV-2 to be higher as a consequence of their chronic illness (question A2), and they thought PD therapy increased their risk of being affected by the virus (question A7). They also believed that the epidemic prevented them from receiving adequate neurological therapies (question A5). Finally, they considered the information provided by newspapers, social media, and mass media about the COVID-19 epidemic as adequate (question A4).

### 3.2. Section B—Personal Experience and Involvement in the Epidemic

This second section was completed by 276 individuals. In our population, 7.97% of patients reported symptoms related to a possible COVID-19 infection (question B1), such as fever (60%), cough (40%), dyspnoea (20%), and others (60%) (question B2). Only a small number of those who reported possible COVID-19-related symptoms in question B1 received home health care services (16.67%, question B3.1), mainly by their general practitioner. A total of 32 participants out of 276 (question B3.2) had a nasal or oropharyngeal swab performed, and, among them, 7 had a positive result (question B3.2.1). Finally, seven patients were hospitalized, but none of them were in intensive care units (question B3.4).

The vast majority of patients did not report substantial difficulties in finding food or drugs during the lockdown period, while face masks were quite hard to acquire (question B4.4: harder or much harder than usual 53.62%).

However, contacting general practitioners was perceived as harder than usual (question B5.1.1). Similarly, PD patients found it difficult to contact their referring neurologists (question B5.2.1). However, these difficulties did not seem to broadly affect the patients’ health conditions (question B5.1.2: no 93.21%, question B5.2.2: no 77.22%).

The health emergency caused restrictions on daily activities. When asked whether they engaged in daily activities as usual, more than 47% of participants answered negatively (question B6.1: much less and less than usual 47.1%). PD patients reduced their physical activities (question B6.2: much less and less than usual 77.53%) and physical therapies (question B6.3: as usual 13.41, not as usual 65.58%). Moreover, a significant part of patients reported therapy to be less effective on PD symptoms (question B6.4: much less and less than usual 44.93%).

### 3.3. Section C—Current Situation

This third section was completed by 260 individuals. The 11 items of section C, in contrast with those in section B, deal with living conditions and daily living at the time of taking the survey, when the lockdown was already over. Results from this section are listed in the following Table 3, side to side with related results from section B.

In comparison with previous months, food, drugs, and face masks were easier to find (items from C1.1 to C1.4), and contacting general practitioners and neurologists was easier as well (items C1.5 and C1.6). A total of 63.46% of individuals returned to their normal daily activities (item C2.1), but less than half of them returned to their previous physical activities (item C2.2), including physical therapies (C2.4). In 32.69% of patients, Parkinson’s disease symptoms were harder to control than they were before the pandemic, even after lockdown restrictions were lifted (question C2.3).

### 3.4. Section D—Personal Data

This fourth section was completed by 246 individuals. This part of our study population was composed of 117 females (47.56%) and 129 males (52.44%) with a mean age of 67.1 years (SD ± 10.3 years) (question D8), while the average age at diagnosis of PD was 58.0 years (SD ± 11.8 years). A total of 73.98% of these patients were married (question D1), and only 7.32% lived alone (question D2). A total of 80.89% did not work when they took our survey (question D4): most of them were retired (67.89%) (question D6). The majority of the population lived in small–medium towns (5000–20,000 inhabitants, 33.33%; 20,000–100,000, 32.11%) (question D7).

Patients were also asked to define their self-sufficiency in activities of daily living. A total of 30.49% declared themselves as self-sufficient, 37.80% as quite autonomous with the sporadic need for help from their caregivers, 25.61% as dependent on their families in daily activities, while 6.10% needed professional assistance (question D9).

## 4. Discussion

This survey-based study aims at evaluating the impact of the COVID-19 pandemic on a population of Italian PD patients. Participation was voluntary and offered through social media posts, by different patients’ associations, and by clinical neurologists during scheduled visits. As mentioned in the previously presented results, every participant had the possibility to leave the survey without completing all the sections; thus, results for each question refer to a specific subpopulation.

Most participants appeared to be worried about the current pandemic, even after the termination of the lockdown period, with over 58% of them thinking PD itself might cause a higher risk for infection, while less than 30% attributed said risk to anti-Parkinson therapies. More than 82% of survey participants were concerned about being in crowded places, including hospitals and ambulatories (more than 72%). These results confirm similar ones obtained by Cavallieri et al., who report a widespread fear among PD patients about the pandemic [2]. A little number of survey participants, however, reported experiencing symptoms possibly related to a SARS-CoV-2 infection (22, 7.97%), with 7 patients (2.5%) who tested positive for SARS-CoV-2 and none of them needing admission to an intensive care unit. In a survey carried out in April 2020, Piano et al. report only slightly higher percentages of possibly COVID-19 related symptoms in the screened PD subpopulation (up to 12%), with no positive tests [15]. Drugs availability during lockdown is reported to be an issue by a limited, but significant, group of our participants (9% for anti-Parkinson drugs, 8% for other drugs), confirming similar findings in the study by Piano et al., in which PD patients were anyway affected the most in comparison with other patients with chronic neurologic diseases [15].

Quite interestingly, a certain overlap exists between participants who felt anti-Parkinson therapies increased their risk of contracting COVID-19 and those who believed the pandemic to adversely affect the availability of proper neurological care. This might imply a role for the patient’s beliefs, in addition to practical difficulties in contacting their primary care physicians and neurologists, in delaying access to counselling and hindering its efficacy. Primary care was indeed quite heavily affected, with about four in ten participants reporting access to such service to be harder or much harder than usual; however, this was rarely identified as a cause for health issues. The impact of lockdown rules was apparently heavier on specialistic care, with more than four in ten participants reporting that it was harder or much harder to obtain a specialistic visit, which in their opinion originated health issues in more than one in five cases. Indeed, while our results show these difficulties did not have a broad effect on the patients’ health conditions, most of the participants who reported issues with PD symptoms worsening and control and who needed adjustments to anti-Parkinson therapy also found it harder to obtain specialistic counselling.

Although most patients were unaffected by the SARS-CoV-2 pandemic per se, the lockdown period itself caused restrictions in everyday activities and especially in physical activity in a large fraction of our sample. Even access to physical therapy was hindered for most patients, and all these restrictions resulted in worse symptoms control for a significant part of our sample during the lockdown period.

Previous studies indeed support our findings on these topics, which highlight an unmet need for specialistic counselling and care in the PD patients’ population, especially among subjects who reported symptoms worsening during lockdown [2]. The decrease in physical activities and the suspension of hospital treatments, including physiotherapy, was already shown to correlate with a perceived subjective worsening of neurological symptoms [15].

Maybe the most interesting result of our survey comes from the comparison between the situation during the lockdown and after lockdown restrictions were lifted. While, after lockdown ended, almost all participants reported no issues with food, drugs and even face masks availability, a significant part of our sample still found it harder than usual to obtain both primary care advice and neurologic counselling. Furthermore, daily activities, physical activities, symptoms control and access to physical therapies were still, in large part, worse than in the pre-pandemic situation.

Taking into account our findings along with our experience with healthcare reorganization during and after the lockdown period [16], we believe that the risk–benefit ratio of limiting healthcare accessibility for chronic diseases (and PD in particular) should be carefully weighed. While the pandemic started as an emergency and resources were forcefully diverted to try and contain the SARS-CoV-2 epidemic, we foresee that both decision-makers and healthcare providers should strive to restore a lost balance between the patients’ need for safety and their rightful demand for readily available services. In this context, telemedicine represents a significant resource, at least for keeping track of disease progression in patients already noted for follow-up [17]. Nonetheless, it is necessary that decision-makers consider a margin for unforeseen healthcare interventions.

## 5. Conclusions

For patients with chronic diseases and even more for Parkinson’s disease patients, who largely benefit from a multi-disciplinary therapeutic approach [15], the restrictions introduced with the lockdown period caused the disruption of a precarious and delicate balance between the patients’ need for continuous and readily available healthcare and overall ability to provide services and counselling, as well as access to dedicated structures for personal physical activities. This balance is still far from being restored, even after months since stricter lockdown restrictions were lifted.

In a population only marginally affected by COVID-19 as a disease, our results show a substantial and durable impact on different aspects of daily life that is heavily influenced by impaired access to health services, which should be carefully considered in the assessment of global health care strategies to overcome the current pandemic and its broader effects.

## Figures and Tables

**Table 1:** English translation of questionnaire items used in the survey.

Code	Question in the Survey
A1	Do you feel worried about the SARS-CoV-2 epidemic?
A2	Do you think your risk of being infected is worsened by your disease?
A3	Are you worried about social distancing and being in crowded places?
A4	What is your opinion about information on the pandemic provided by newspapers, TV, social media?
A5	Do you fear the epidemic will hinder your access to healthcare for your disease?
A6	Are you afraid of going in places such as ambulatories and hospitals?
A7	Do you think your risk of being infected is worsened by the medications you take?
A8	Do you think effective therapies are available for the SARS-CoV-2 infection?
B1	During the 2019/2020 SARS-CoV-2 outbreak, have you suffered from symptoms suggestive of a possible infection?
B2	If that was the case, list your symptoms.
B3.1	Did you receive health care at home?
B3.2	Were you tested with one or more nasopharyngeal swabs?
B3.2.1	Were such tests positive?
B3.3	Were you quarantined at home?
B3.4	Were you admitted to the hospital?
B4.1	During the lockdown period, did you find it harder to get food?
B4.2	During the lockdown period, did you find it harder to obtain antiparkinsonian drugs?
B4.3	During the lockdown period, did you find it harder to obtain other drugs?
B4.4	During the lockdown period, did you find it harder to obtain face masks?
B5.1.1	During the lockdown period, did you find it harder to make contact with your primary care physician?
B5.1.2	In case you found it harder, were there any consequences on your health?
B5.2.1	During the lockdown period, did you find it harder to make contact with your neurologist?
B5.2.2	In case you found it harder, were there any consequences on your health?
B6.1	During the lockdown period, you engaged in daily activities as usual:
B6.2	During the lockdown period, you engaged in motor activity as usual:
B6.3	During the lockdown period, you received physical therapies as usual:
B6.4	During the lockdown period, symptoms control was the same as before:
B7	Use this space to freely write any comments on your experience during lockdown (from 9 March 2020 to 18 May 2020):
C1.1	In the current situation, is obtaining food still harder than before lockdown?
C1.2	In the current situation, is obtaining antiparkinsonian drugs still harder than before lockdown?
C1.3	In the current situation, is obtaining other drugs still harder than before lockdown?
C1.4	In the current situation, is obtaining face masks still harder than before lockdown?
C1.5	In the current situation, is contacting your primary care physician still harder than before lockdown?
C1.6	In the current situation, is contacting your neurologist still harder than before lockdown?
C2.1	Is your coping with daily activities back to normal?
C2.2	Is your engagement in motor activities back to normal?
C2.3	Is symptoms control back to normal after the lockdown period?
C2.4	Are you receiving physical therapies as usual, after the lockdown period?
C3	Use this space to freely write any comments on your experience in the current situation:
Sex	Select your sex:
Age	State your age:
D1	What is your marital status?
D2	Do you live with other persons?
D3	What is your education level?
D4	Are you currently working?
D5	Select your contract type:
D6	Select your work type:
D7	Select the size of your town or city:
D8	At what age were you diagnosed with Parkinson?
D9	Are you self-sufficient?

**Table 2:** Results from section A—opinion and perception of the epidemic.

Item	Not at All	Slightly	Moderately	Very	Extremely
A1	5 (1.47%)	44 (12.98%)	162 (47.79%)	102 (30.09%)	26 (7.67%)
A2	42 (12.39%)	98 (28.91%)	124 (36.58%)	56 (16.52%)	19 (5.6%)
A3	4 (1.18%)	52 (15.34%)	154 (45.43%)	89 (26.25%)	40 (11.8%)
A4	**Totally** **inadequate**	**Inadequate**	**Adequate**	**Excessive**	**Overly** **excessive**
	12 (3.54%)	49 (14.45%)	148 (43.66%)	112 (33.04%)	18 (5.31%)
	**Not at all**	**Slightly**	**Moderately**	**Very**	**Extremely**
A5	33 (9.73%)	77 (22.71%)	103 (30.38%)	81 (23.89%)	45 (13.27%)
A6	23 (6.78%)	63 (18.58%)	127 (37.46%)	84 (24.78%)	42 (12.39%)
A7	109 (32.15%)	131 (38.64%)	71 (20.94%)	21 (6.19%)	7 (2.06%)
A8	**No**		**Maybe**	**Yes**	
	135 (39.82%)		167 (49.26%)	37 (10.91%)	

**Table 3:** Side to side results from section B—Personal Experience and Involvement in the Epidemic and section C—Current situation.

Thematic	Experience in the Pandemic (n = 276)						Current Situation (n = 260)		
	Item	Much Easier than Usual	Easier than Usual	Same	Harder than Usual	Much Harder than Usual	Item	Still Harder	Not Still Harder
Food	B4.1	2 (0.72%)	2 (0.72%)	187 (67.75%)	73 (26.45%)	12 (4.35%)	C1.1	8 (3.08%)	252 (96.92%)
Parkinson drugs	B4.2	5 (1.81%)	1 (0.36%)	245 (88.77%)	22 (7.97%)	3 (1.09%)	C1.2	5 (1.92%)	255 (98.08%)
Other drugs	B4.3	4 (1.45%)	3 (1.09%)	247 (89.49%)	18 (6.52%)	4 (1.45%)	C1.3	5 (1.92%)	255 (98.08%)
Face masks	B4.4	3 (1.09%)	4 (1.45%)	121 (43.84%)	97 (35.14%)	51 (18.48%)	C1.4	8 (3.08%)	252 (96.92%)
Primary care	B5.1.1	2 (0.72%)	7 (2.54%)	158 (57.25%)	90 (32.61%)	19 (6.88%)	C1.5	50 (19.23%)	210 (80.77%)
Neurologist	B5.2.1	2 (0.72%)	3 (1.09%)	150 (54.35%)	82 (29.71%)	39 (14.13%)	C1.6	58 (22.31%)	202 (77.69%)
		**No, much less**	**No, less**	**Yes**	**More than usual**	**Much more than usual**		**Back to normal**	**Not back to normal**
Daily activities	B6.1	47 (17.03%)	83 (30.07%)	130 (47.1%)	15 (5.43%)	1 (0.36%)	C2.1	165 (63.46%)	95 (36.54%)
Motor activity	B6.2	105 (38.04%)	109 (39.49%)	55 (19.93%)	6 (2.17%)	1 (0.36%)	C2.2	125 (48.08%)	135 (51.92%)
		**Yes**	**No**		**I never did**				
Physical therapies	B6.3	37 (13.41%)	181 (65.58%)		58 (21.01%)		C2.3	175 (67.31%)	85 (32.69%)
		**No, much less**	**No, less**	**Yes**	**More than usual**	**Much more than usual**			
Symptoms control	B6.4	21 (7.61%)	103 (37.32%)	149 (53.99%)	3 (1.09%)	0 (0%)	C2.4	90 (44.12%)	114 (55.88%)

## Data Availability

The data presented in this study are available in Supplementary Material 1: Survey data.

## Supplementary Materials

The following supporting information can be downloaded at: https://www.mdpi.com/article/10.3390/jcm11113007/s1, Supplementary Material 1: Survey data.
